# Antiproliferative and Antiestrogenic Activities of Bonediol an Alkyl Catechol from *Bonellia macrocarpa*

**DOI:** 10.1155/2015/847457

**Published:** 2015-10-18

**Authors:** Rosa Moo-Puc, Edgar Caamal-Fuentes, Sergio R. Peraza-Sánchez, Anna Slusarz, Glenn Jackson, Sara K. Drenkhahn, Dennis B. Lubahn

**Affiliations:** ^1^Unidad de Investigación Médica Yucatán, Unidad Médica de Alta Especialidad, Centro Médico Ignacio García Téllez, Instituto Mexicano del Seguro Social (IMSS), Calle 41 No. 439, Colonia Industrial, 97150 Mérida, YUC, Mexico; ^2^Unidad de Biotecnología, Centro de Investigación Científica de Yucatán (CICY), Calle 43 No. 130, Colonia Chuburná de Hidalgo, 97200 Mérida, YUC, Mexico; ^3^Department of Biochemistry, University of Missouri, 117 Schweitzer Hall, Columbia, MO 65211, USA; ^4^Nebraska College of Technical Agriculture Veterinary Technology Program, 404 East 7th Street, Curtis, NE 69025, USA; ^5^Lindenwood University Belleville, 2600 W. Main, Belleville, IL 62226, USA

## Abstract

The purpose of this study was to investigate antiproliferative activity of bonediol, an alkyl catechol isolated from the Mayan medicinal plant *Bonellia macrocarpa*. Bonediol was assessed for growth inhibition of androgen-sensitive (LNCaP), androgen-insensitive (PC-3), and metastatic androgen-insensitive (PC-3M) human prostate tumor cells; toxicity on normal cell line (HEK 293) was also evaluated. Hedgehog pathway was evaluated and competitive 3H-estradiol ligand binding assay was performed. Additionally, antioxidant activity on Nrf2-ARE pathway was evaluated. Bonediol induced a growth inhibition on prostate cancer cell lines (IC_50_ from 8.5 to 20.6 *µ*M). Interestingly, bonediol binds to both estrogen receptors (ER*α* (2.5 *µ*M) and ER*β* (2.1 *µ*M)) and displaces the native ligand E2 (17*β*-estradiol). No significant activity was found in the Hedgehog pathway. Additionally, activity of bonediol on Nrf2-ARE pathway suggested that bonediol could induce oxidative stress and activation of detoxification enzymes at 1 *µ*M (3.8-fold). We propose that the compound bonediol may serve as a potential chemopreventive treatment with therapeutic potential against prostate cancer.

## 1. Introduction

Prostate cancer is the second most frequently diagnosed cancer and the sixth leading cause of cancer death in males. Approximately, one man in five will be diagnosed with prostate cancer during his lifetime, and 1 man in 33 will die of this disease [1]. Treatment for this disease may include radiation therapy and androgen suppression; surgery and/or chemotherapy are often used. However, adverse effects have been described, decreasing the quality of life of patients [2]. Furthermore, in 15% of the patients, the cancer recurs within a few years as an advanced “hormone-refractory” and often metastatic disease. For these patients, there are few treatment options available [3], and the 5-year survival rate decreases to 28% [4].

Chemotherapy is a good option for the treatment of hormone-independent and hormone-dependent prostate cancer; however, few therapies in clinical phase of development are available [5]. In addition, some cancers have proven to be resistant to chemotherapy drugs [6]. Therefore, identification of new drugs for prostate cancer treatment has significant clinical implications.

Currently one of the signaling pathways that has been of great interest because of its importance in the development and progression of prostate cancer is the sonic Hedgehog (Shh) signaling pathway. The Shh pathway involves the autocleavage of full length Shh into an active 20 kD N-terminal fragment (ShhN), which binds to its 12-pass transmembrane receptor, Patched (Ptc1), reversing (relieving) its inhibitory effect on Smoothened (Smo). In prostate cancer, Shh pathway can produce malignant transformation of primitive prostate epithelial progenitor cells; this may be initiated by trapping of a normal stem cell in a Shh-dependent state of continuous renewal, which promotes tumor growth [7–10].

The estrogen and Hedgehog signaling pathways are crucial for physiological proliferation, differentiation, and development of the mammary and prostate glands [11, 12]. It has also been found that activation of both Shh and ER*α* can lead to the growth of cancerous tumors (insert references here that show Shh and ERa in breast and prostate cancer). Moreover studies suggest that ER*α* regulates the Shh pathway and promotes cancer development [13–17]. Recently a study using* in vitro* and* in vivo* models suggested estrogen, mediated through ER*α* and ER*β*, could induce carcinogenesis and various types of toxicity in a normal prostate [18]. Investigations searching for new compounds that can inhibit the Hedgehog pathway and regulate the ERs to treat or prevent prostate cancer could have significant implications [19, 20].

A direct relationship between an increase in reactive oxygen species (ROS) and the induction of the Shh pathway has also been documented. This induction promotes the expression of the antiapoptotic gene Bcl-2 and inhibits the expression of the proapoptotic gene Bax [21]. In addition, Paschos et al. [22] suggest that androgens and estrogen play an important role in the generation of reactive oxygen species leading to the progression of prostate cancer and that the antioxidant activity of certain small molecules may prevent the progression of prostate cancer. NF-E2 Related Factor 2 (Nfr2) is an important transcription factor responsible for stimulating the transcription of genes in response to oxidative or electrophilic stress [23]. This process involves the binding of Nrf2 with Maf protein in the nucleus to form a heterodimer, subsequently interacting with antioxidant responsive element (ARE) to activate gene transcription [24]. The Nfr2-ARE pathway induces transcription of antioxidant proteins and phase II detoxifying enzymes, which are important for protection of cells against ROS damage [23]. Thus this pathway may serve as a marker of oxidative stress damage. The three ERs family members, ER*α*, ER*β*, and ER*γ*, play a novel functional role in the inhibitor of Nrf2 transcriptional activity. It is also the modulation of ER*α* and ER*β* that may be useful as a therapeutic target in cancer chemoprevention studies or for the development of selective estrogen receptor modulators with a lower risk of causing cancer [25–28].

In our continuous effort to search for novel anticancer agents from Mayan medicinal plants of the Yucatan peninsula, we recently isolated a novel compound from the medicinal plant* Bonellia macrocarpa* (Cavanilles) Ståhl and Källersjö. This new alkyl catechol, called bonediol, has been demonstrated to have interesting antiproliferative activities* in vitro* on cancer cell lines [29]. Accordingly, this study evaluated the antiproliferative properties of bonediol in various lines of prostate cancer and also explored its effect on the Shh signaling pathway, interactions with the Nrf2 antioxidant response element, and potential binding to estrogen receptors (ER*α* and ER*β*).

## 2. Material and Methods

### 2.1. Isolation of Bonediol

Root bark of* B. macrocarpa* was collected from Telchac Puerto, Yucatan (Mexico). The plant material was identified and authenticated by taxonomists from the Department of Natural Resources of the Scientific Research Center of Yucatan (CICY). Specimens under the voucher number P. Simá 2979 were deposited at CICY's U Najil Tikin Xiw herbarium. The obtaining and characterization of the compound were performed as previously described [29]. The pure compound was dissolved in DMSO and stored at −20°C.

### 2.2. Cell Culture

Cell lines of human prostate cancer adenocarcinoma (PC-3), a metastatic variant of PC-3 (PC-3M), hormone sensitive human prostate carcinoma (LNCaP), and one normal human cell line (HEK-293) were obtained from the American Type Culture Collection (ATCC). Shh Light II (JHU-68) and COS-1 cells lines were used to evaluate Shh and Nrf2-ARE pathways, respectively. The cells line PC-3 was propagated in F-12K medium (Gibco) and LNCaP in RPMI-1640 medium (Gibco). COS-1, HEK-293, and PC-3M cells lines were propagated in Dulbecco's modified Eagle's medium (DMEM) (Gibco). Shh Light II cells were maintained in DMEM containing 4 mmol/L of L-glutamine adjusted with 1.5 g/L sodium bicarbonate and 4.5 g/L glucose, supplemented with 0.4 mg/mL G-418 and 0.15 mg/mL zeocin (Invitrogen). All cell lines were cultured in sterile Costar T75 flasks containing fetal bovine serum (10% v/v), 100 U/mL penicillin G, and 100 mg/mL streptomycin at 37°C under a humid atmosphere containing 5% CO_2_.

### 2.3. Antiproliferative Activity

Cells were cultured in 96-well plates at a concentration of 5 × 10^4^ cells per well; after being cultured for 24 h at 37°C in an atmosphere of 5% CO_2_ (95% humidity) cells were incubated with appropriate dilutions of the test compound for 48 h. The growth inhibition of the cell lines was evaluated by the sulforhodamine B method [30]. Results are expressed as the concentration of agent that reduces cell growth by 50% (IC_50_). Docetaxel was used as a positive control. All determinations were performed in triplicate. In addition, the degree of toxicity to normal cells was evaluated, by determining the selectivity index (SI) [31].

### 2.4. Assay of Inhibition from Hedgehog Pathway

Gli activity in the Shh Light II cell line was assayed after 48 h of treatment with bonediol compound in phenol red-free DMEM supplemented with 0.5% charcoal-stripped serum using the Dual Luciferase Reporter Assay System (Promega). Each experiment was performed at least thrice in duplicate. Mouse recombinant Shh was obtained from R&D Systems. Shh was dissolved in PBS with 0.1% bovine serum albumin. In each experiment, the controls and all treatments contained all vehicles used. All treatments were conducted in phenol red-free medium with charcoal-stripped serum [32]. Each experiment was performed at least thrice in duplicate.

### 2.5. Competitive Binding Assay

Proteins were synthesized using the TNT Coupled Reticulocyte Lysate System from Promega.* In vitro* transcription/translation products were treated individually with various concentrations of [3H]-17-*β*-E2 in the absence or presence of various doses of unlabeled competitors or unlabeled 17-*β*-E2 overnight at 4°C in order to achieve equilibrium binding. Bound and free ligand were separated by dextran-coated charcoal. Relative binding affinity (RBA) was determined by dividing the IC_50_ of the unlabeled 17-*β*-E2 by the IC_50_ of the unlabeled competitor.

### 2.6. Plasmid

The vectors containing Gal4-luciferase, the 4x mouse GST Ya subunit ARE (4 copies of the 41 bp GST Ya element) reporter, hemagglutinin (HA) tagged Nrf2, and the Nrf2 transactivation domain-Gal4 DNA binding domain fusion vector have been described previously [25, 28, 33]. phRG-TK control renilla luciferase vector was obtained from Promega.

### 2.7. Regulation of Antioxidant Response Element (Nrf2-ARE) Assay

The transcriptional activity of NF-E2 Related Factor 2 (Nrf2) on antioxidant response element (ARE) was monitored as previously described [25]. Briefly, COS-1 cells were seeded in 24-well plates in phenol red-free medium with 10% dextran-coated charcoal-stripped fetal bovine serum, for transient transfection with 150 ng 4x mouse GST Ya subunit ARE firefly luciferase reporter or Gal4-luciferase reporter, 10 ngphRGTK control renilla luciferase vector, and different expression vectors using plus and Lipofectamine reagents (Invitrogen, Carlsbad, CA). Constant transfected DNA amount was compensated by empty vector—pcDNA3.1(+)zeo (Invitrogen, Carlsbad, CA). After 12–16 h, transfected cells were then treated with bonediol or vehicle. After 24 h of incubation, cells were rinsed with PBS twice and lysed to measure the luciferase level using the Dual Luciferase assay kit (Promega, Madison, WI). Data were normalized to the cotransfected phRG-TK control renilla luciferase activity. All experiments were performed at least three times with duplicate samples per experiment.

### 2.8. Statistical Analysis

Graph Pad Prism 4 (Graph Pad Software, La Jolla, CA) was used to calculate *P* values of *P* < 0.05 which were considered significant in all cases. The IC_50_ were calculated using doses-response nonlinear fit curve. One-way analysis of variance (ANOVA) was used to assess significant differences among treated groups followed by Dunnett's test.

## 3. Results

**Figure 1:**
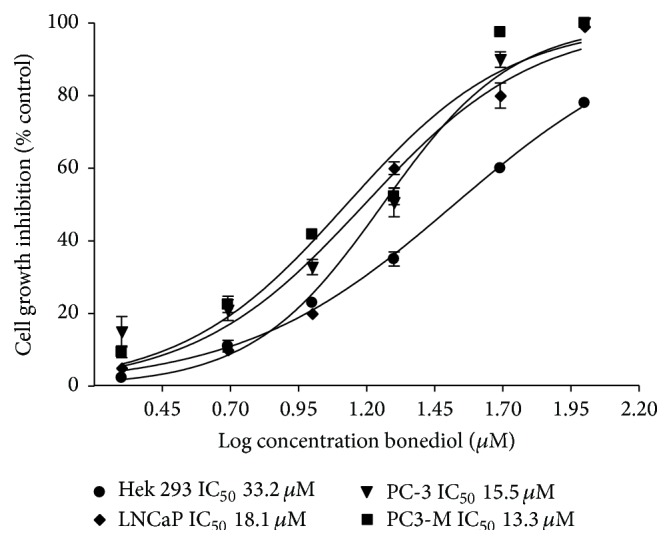
Bonediol inhibits cell growth on HEK-293, LNCaP, PC-3, and PC-3M cells. Various concentrations of bonediol were used for 48 h and the effects were examined using SRB colorimetric assay. Each experiment was performed at least thrice in duplicate. IC_50_ values represent the concentration of the compound at which half-maximal inhibition we observed.

In order to explore the possible antiproliferative effect of the compound bonediol, a SRB assay was performed to determine whether this molecule was able to inhibit the growth of prostate cancer cells. A typical dose-response behavior was observed in all cell lines tested, with IC_50_ in the various cell lines tested ranging from 8.5 to 20.6 *μ*M (Figure 1).  Table 1 shows the median concentration that inhibited cell growth (IC_50_) and the selectivity index of bonediol towards all cell lines. Bonediol inhibited the growth of PC-3, LNCaP, and metastatic PC-3M cell lines with selectivity compared with Hek-293.

Shh Light II cell, an NIH 3T3 cell line stably transfected with Gli1-dependent firefly luciferase and constitutive renilla luciferase reporters, was used to explore the ability of bonediol to inhibit Shh pathway activation. When we tested the model with various concentrations of bonediol (0.1, 0.5, 1, and 5 *μ*M) no significant inhibition was observed compared with the control cyclopamine (data not shown).

**Figure 2:**
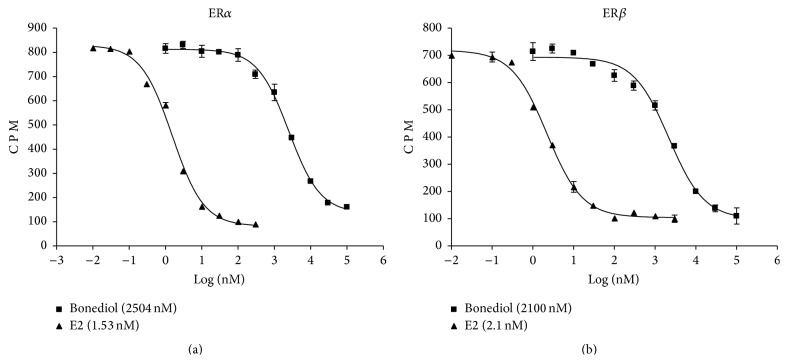
Dose-response curves of 17*β*-estradiol and bonediol in the radio ligand receptor binding assay using [3H]17b-estradiol and human estrogen receptor expressed in TNT Coupled Reticulocyte Lysate System: (a) ER*α* and (b) ER*β*.

We next analyzed the potential of bonediol to bind to estrogen receptors (ER*α* and ER*β*) (Figure 2). Bonediol binds to both receptors in a dose-dependent manner; 2.5 *μ*M and 2.1 *μ*M displaced 50% of estradiol binding on ER*α* and ER*β*, respectively.

**Figure 3:**
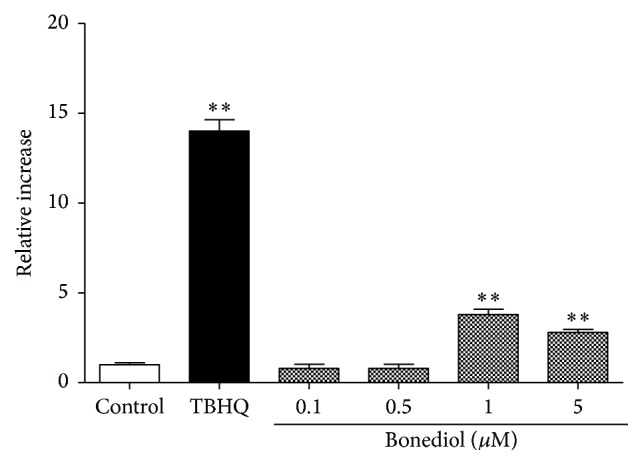
Bonediol activate NRF2-ARE. Relative Nrf2-ARE activation from three assays is normalized with the control group. Each symbol is the mean ± SD. ^*∗∗*^ *P* < 0.01 versus control.

As mentioned above, the activation of the Nrf2 signaling pathway governs the expression of ARE-driven genes. This pathway has been associated with induction of oxidative stress and in this study was used as a possible marker of oxidative damage from bonediol to the cells. Bonediol induces activation of Nrf2-ARE in transfected COS-1 cells at 1 *μ*M (3.8-fold) and 5 *μ*M (2.8-fold) (Figure 3). The results show that bonediol activates Nrf2-ARE signaling possibly through induction of oxidative stress.

## 4. Discussion

This study evaluated for the first time the activity of bonediol on binding to the estrogen receptors, inhibition of the Shh pathway, and activation of Nrf2-ARE signaling. Previous work demonstrated the potential antiproliferative effect of bonediol [29] and that this compound has no significant cytotoxic activity [34]. Furthermore, bonediol does not induce apoptosis at low doses [34]. We explored here the antiproliferative potential of bonediol against three prostate cancer lines (hormone sensitive (LNCaP), hormone insensitive (PC-3), and high metastatic hormone insensitive PC-3M) and the possible pharmacologic mechanisms. Bonediol has an antiproliferative effect in the three prostate cancer cell lines, indicating that this compound could have various pharmacologic mechanisms, both hormone-dependent and hormone-independent. Prostate cancer has been reported to have an altered Shh signaling as a pathway of importance in advanced growth and this pathway has recently been shown of interest in the search for new compounds that can inhibit cancer [32, 35]. However, bonediol did not demonstrate an inhibition of this signaling pathway to a concentration of 10 *μ*M (data not shown). No further evaluations were performed at doses, since we observed damage to the cells without concomitant inhibition of Shh signaling. Interestingly, bonediol displaced 50% of estradiol on ER*α* and ER*β* at concentrations of 2.1 and 2.5 *μ*M, respectively. No studies on the estrogenic potential of alkyl catechols have been reported previously, but there are reports of related synthetic alkyl phenols compounds that have antiestrogenic effect [36]. Furthermore, some studies indicate that the main pharmacophore for recognition by the estrogen receptor is the presence of at least one phenolic alcohol and hydrophobic long chain [37, 38]. We do not know precisely how bonediol is binding to the ERs, but the presence of the two phenolic alcohols and long chain hydrophobic may be involved. The binding of the compound bonediol to the ERs does not indicate whether bonediol could be acting as an antagonist or as an agonist. Further studies are required to observe the way in which this compound could regulate either receptor. It is known that preferential ER*β* activation has an antiproliferative effect in breast and prostate cells and is viewed as a protective balance against ER*α* activation, which is associated with proliferation [39–41]. Bonediol may be regulating both ER*α* and ER*β*, resulting in an antiproliferative effect on prostate cancer. Moreover, Nelles et al. [19] remarked the importance of development of new selective ER modulators with therapeutic potential.

Finally, we found that bonediol activates Nrf2-ARE signaling at a concentration of 1 *μ*M (3.8-fold induction), which is indicative of oxidative stress and may be a mechanism of damage to the cell lines tested. However, another explanation for the activation of this pathway could be that some chemical compounds with antioxidant properties have the ability to be redox active and activate the Nrf2-ARE pathway [42]. In this context, some related alkyl phenols have shown antioxidant and prooxidant activity [43, 44]. Antioxidants from plants have been studied for the prevention of several cancer types, including prostate cancer, and it is currently believed that small doses of these compounds could have a beneficial effect by inducing the activation of antioxidant proteins and detoxifying enzymes, which would act against carcinogenic insults [23]. At a concentration of 5 *μ*M bonediol had a slightly lower induction (2.8-fold), compared with the inductive effect at 1 *μ*M (3.8-fold). This effect could be due to the compound exhibiting toxic effects in cell lines at the higher concentration.

In the course of our research we found that the compound bonediol is able to bind ER*α* and ER*β*. This is the first report of the potential estrogenic activity of these compounds (alkyl catecohols), isolated from plants. Additionally, bonediol induces activation of Nrf2-ARE, possibly functioning as an antioxidant and generating oxidative stress.

Future studies aimed at elucidating how this compound binds to the estrogen receptors and the exact mechanism by which bonediol activates Nrf2-ARE will be beneficial to development of a potential new prostate cancer therapeutic.

## 5. Conclusion

In summary, we found that bonediol binds to both ER*α* and ER*β* in the low micromolar range, has potential estrogenic activity, and can induce Nrf2 signaling. Furthermore, we propose that the compound bonediol may serve as a potential chemopreventive treatment with therapeutic potential against prostate cancer.

## Figures and Tables

**Table 1:** Antiproliferative activity IC_50_ (*µ*M) and selective index of bonediol from *B. macrocarpa*.

Compound	Cell lines IC_50_ *μ*M (SI)			
	Hek-293	LNCaP	PC-3	PC3-M
Bonediol	33.2	18.1 (1.8)	15.5 (2.1)	13.3 (2.5)
Docetaxel	1.10	0.23 (4.78)	0.20 (5.50)	0.08 (13.75)
